# Investigating the Role of Loop C Hydrophilic Residue ‘T244’ in the Binding Site of ρ1 GABA_C_ Receptors via Site Mutation and Partial Agonism

**DOI:** 10.1371/journal.pone.0156618

**Published:** 2016-05-31

**Authors:** Moawiah M. Naffaa, Nathan Absalom, V. Raja Solomon, Mary Chebib, David E. Hibbs, Jane R. Hanrahan

**Affiliations:** Faculty of Pharmacy, University of Sydney, Sydney, NSW, Australia; Danish Cancer Society Research Center, DENMARK

## Abstract

The loop C hydrophilic residue, threonine 244 lines the orthosteric binding site of ρ1 GABA_C_ receptors was studied by point mutation into serine, alanine and cysteine, and tested with GABA, some representative partial agonists and antagonists. Thr244 has a hydroxyl group essential for GABA activity that is constrained by the threonine methyl group, orienting it toward the binding site. Significant decreases in activation effects of the studied ligands at ρ1 T244S mutant receptors, suggests a critical role for this residue. Results of aliphatic and heteroaromatic partial agonists demonstrate different pharmacological effects at ρ1 T244S mutant receptors when co-applied with GABA EC_50_ responses. ρ1 T244A and ρ1 T244C mutant receptors have minimal sensitivity to GABA at high mM concentrations, whereas, the ρ1 WT partial agonists, β-alanine and MTSEA demonstrate more efficacy and potency, respectively, than GABA at these mutant receptors. This study explores the role of Thr244 in the binding of agonists as an initial step during channel gating by moving loop C towards the ligand.

## Introduction

The γ-aminobutyric acid type C (GABA_C_) receptors, also known as γ-aminobutyric acid type A rho (ρ GABA_A_) receptors are ligand gated ion channel receptors that activated by GABA, the most inhibitory neurotransmitter substance in the vertebrate CNS (central nervous system) [1]. The rational discovery of selective agents initially revealed two distinct classes of GABA receptors, the ionotropic GABA_A_ and metabotropic GABA_B_ receptors. The two classes are different in their pharmacology, biochemical and electrophysiology properties [2]. A subclass of ligand-gated ion channels were later identified, insensitive to ligands that typically affected GABA_A_ and GABA_B_ receptors, these were initially classed as GABA_C_ receptors [3]. Receptors of the ligand-gated ion channel superfamily are pentameric requiring five subunits to assemble a single ion channel. The ion channel may be homomeric, formed by five identical subunits as is the case for ρ GABA_C_ receptors or heteromeric, formed by a combination of at least two different subunits, such as the GABA_A_ receptors [4].

GABA_C_ ion channels expressed in *Xenopus* oocytes have different properties to GABA_A_ ion channels in terms of potency, channel opening time and receptor desensitization. In general, GABA is between ten- and one hundred-fold more potent at GABA_C_ receptors than at GABA_A_ receptors, with slow activation and deactivation, and less readily desensitized [5–7]. The pharmacological profile of GABA_C_ receptors is distinguished by many ligands that are selective at this subfamily over GABA_A_ receptors.

The novel ρ1 GABA_C_ subunit from a human retina cDNA library was cloned in the early 1990s [8]. The second member of this subfamily, designed ρ2 was cloned from human retina a year later [9]. A third member of this subfamily designed ρ3 has also been detected in retina as well as higher brain regions with lower expression levels compared with the retinal level [10, 11]. In the retina, ρ3 is found expressed in ganglion neurons of the retina while ρ1 and ρ2 are specifically expressed in bipolar and horizontal cells [12–14].

Many Studies have suggested the involvement of them in the sleep-waking behaviour of rats [15], learning and memory in chicks and rats [16], inhibition of ammonia-induced apoptosis in hippocampal neurons [17], and hormone release in the pituitary [18]. More recently, the GABA_C_ antagonist TPMPA, was shown to improve the symptoms of retinitis pigmentosa in rats [19], and evidence that ρ1 and ρ2 may be important for different specific in vivo effects of ethanol, has been reported [20].

The structure of ρ1 receptors was first studied by identifying critical residues that have direct effects on ligand sensitivity using site-directed mutagenesis and substituted cysteine accessibility methods. The T244 residue in loop C has the hydroxyl side-chain oriented toward the GABA binding site. Mutation of this residue to various amino acids resulted mostly in non-functional receptors even at high concentrations of GABA, and only the T244S mutation resulted in a functional receptor, although GABA potency was decreased 35-fold [21]. Additionally, ρ1 GABA_C_ T244S mutant receptors have recently been studied with various ligands. This mutation resulted in many folds decrease in potency of the studied agonists, whilst the studied antagonists remain unaffected by the mutation [22].

**Fig 1:**
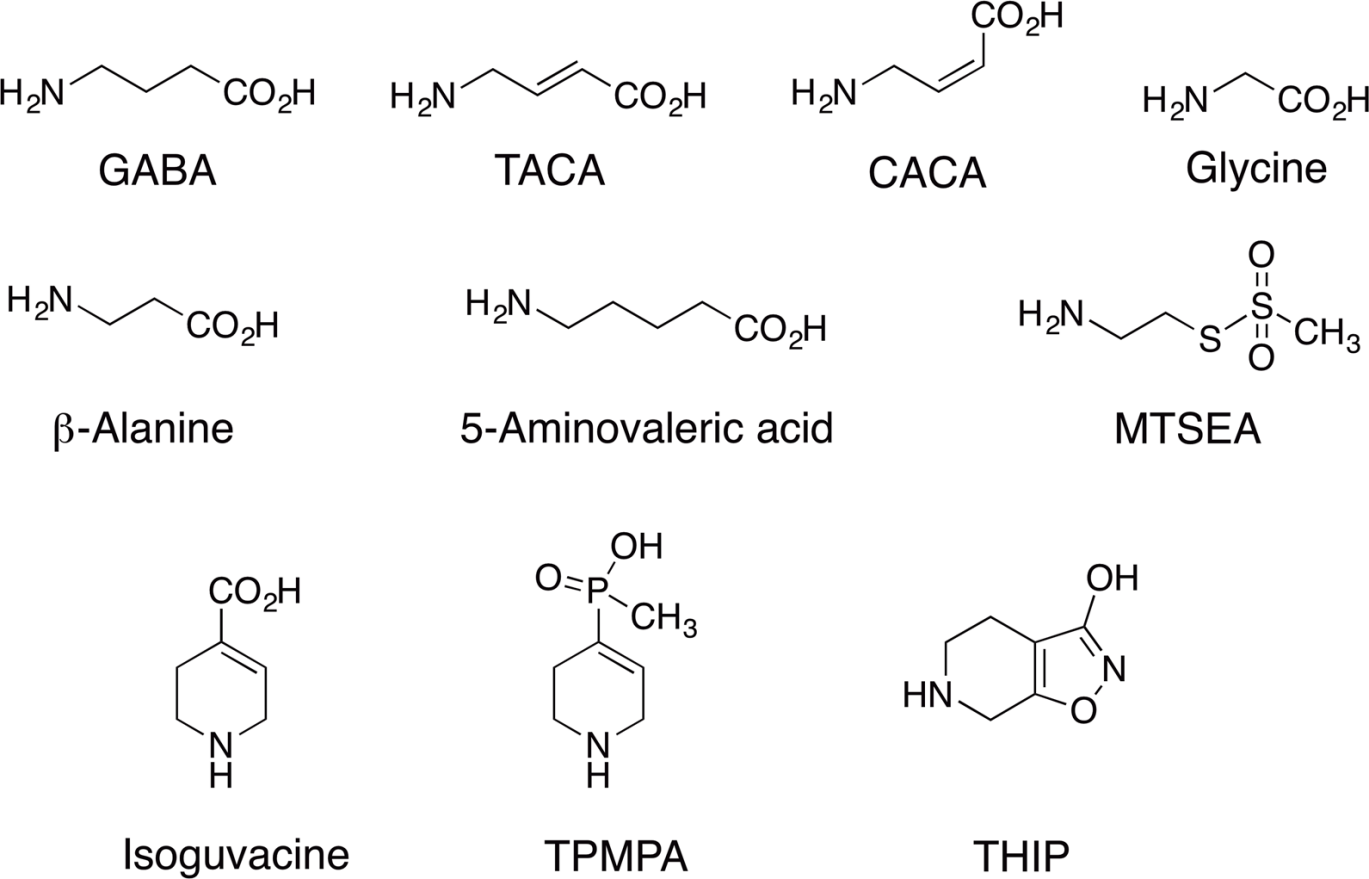
Chemical structures of ligands used in this study.

The current study aims to explore the role of this hydrophilic residue in the orthosteric binding site in terms of receptor functionality, and to determine the interactions it may form with various ligands. Furthermore, the shift in the potency and efficacy of the studied ligands (Fig 1), and additive (or co-operative)/inhibition effects are also considered and discussed in terms of how different interactions may affect the receptor stability in *apo* state or open conformation.

## Method, Experiment and Materials

### Molecular modeling

#### Sequence alignment and homology modelling

The protein sequence of human ρ1 GABA_C_ was obtained from the universal protein resource (http://www.uniprot.org/) [23], and the models were built on templates of the GluCl in open conformation (PDB ID 3RIF) and *apo* state (PDB ID 4TNV) (http://www.rcsb.org/).[24]. Sequence alignment was performed using the CLUSTALW program [25]. The models of the ρ1 GABA_C_ receptor were built using Prime 3.2 software [26]. The preparation, generation, validation and selection of the homology model of the ρ1 GABA_C_ receptors was recently described [27].

### Ligand preparation

The studied ligands in this work were first drawn using the 2D sketcher and then manipulated and adjusted for chemical correctness using Schrödinger’s Maestro (Maestro, v9.9) interface [28]. The preparation of all compounds for docking was performed by LigPrep (LigPrep v3.1) [29].

#### Molecular docking of ligands into the orthosteric binding site

The docking studies of ligands with the generated models were carried out using the default settings of the Glide program in the extra-precision mode implemented in Schrödinger Suite 2013 [30].

Induced fit docking was also performed for the studied ligands in the ρ1 GABA_C_ orthosteric binding site. The full methodology of the docking studies has been previously described in conjunction with the ρ1 GABA_C_ homology model used in this work [27]. For the models of each studied mutant, the studied ligands also underwent induced fit docking into the new binding sites, however no significant changes to the binding site was identified. The orientation of the introduced amino acid side-chains after after induced fit docking of the various ligands were also considered, and other side-chain rotamers also checked for further confirmation of ligand binding and interactions with the introduced residues at Thr244 position.

### Molecular biology

#### Materials

Human ρ1 cDNA subcloned into pcDNA1.1 (Invitrogen, San Diego, CA, USA) was kindly provided by Dr. George Uh1 (National Institute for Drug Abuse, Baltimore, MD, USA).

(GABA, 5-aminovaleric acid, β-alanine, glycine, isoguvacine, 4,5,6,7-tetrahydroisoxazolo[5,4-c]pyridin-3-ol (THIP) also known as Gaboxadol, were purchased from Sigma-Aldrich (Sigm-Aldrich Pty Ltd, Castle Hill, NSW 1765 Australia). 1,2,5,6-Tetrahydropyridin-4-yl)methylphosphinic acid (TPMPA) [31], *trans*-aminocrotonic acid (TACA) and *cis*-aminocrotonic acid (CACA) [32], and 2-aminoethylmethane thiosulfonate (MTSEA) [33] were prepared as previously reported.

#### Primer design and site-directed mutagenesis

Pairs of the complementary mutagenic oligonucleotide primers were first designed to introduce single point mutations at human ρ1 GABA_C_ subunit using UniProt [34]. Primers were made by life technologies (Life Technologies^TM^ Australia Pty Ltd).

Site-directed mutagenesis was performed using QuikChange II site-directed mutagenesis kit as described by the manufacturer (Stratagene) [35, 36].

#### Transformation and preparation of wild type and mutant plasmids

The desired mutant or wild type plasmid DNAs of ρ1 subunit were transferred into TOP10 Chemically Competent *E*. *coli* according to the protocol (Invitrogen™)). The *E*.*Coli* were grown in LB broth with ampicillin (100 μg/ml) then the cell harvesting and the plasmids DNA purification were performed using Qiagen Spin Miniprep Kit (Qiagen, VIC, Australia).

#### In vitro transcription

ρ1 GABA_C_ WT and mutant plasmids DNA were linearized by incubation with Xba1. ρ1 WT and mutant cRNA were synthesized using the T7 Transcription mMESSAGE mMACHINE Kit (Ambion, Austin, TX, USA) following the procedure described by the manufacture. The quantity and quality of synthesized _C_RNA was determined by absorbance at 260–280 nm using Nanodrop Spectrophotometer (Thermo Scientific, Wilmington, DE, USA). The purity of synthesized cRNA was confirmed by agarose gel electrophoresis (0.9%) using the GelDoc 1000 (Bio-Rad Laboratories, Hercules, CA USA).

### Electrophysiology

#### Oocyte preparation and RNA injection

Xenopus laevis are purchased from Nasco in Fort Atkinson, Wisconsin, USA and housed at the University of Sydney Xenopus laevis facility. Frogs are maintained in groups of 18–22°C water at a depth of 12 cm with a 12 hrs light dark cycle, and fed twice weekly. Mature female Xenopus laevis frogs were anaesthetised (0.17% tricaine, buffered with 0.06% sodium bicarbonate) until the loss of righting reflex was confirmed (15 minutes) before transferring them on to ice where surgeries were performed. A small (1–2 cm) incision was made using surgical knives through both the skin and muscle layer of the abdomen. Ovary lobes were removed with a pair of forceps and placed in oocyte releasing 2 (OR2) buffer (82.5 mM NaCl, 2 mM KCl, 1 mM MgCl2, 5 mM HEPES hemisodium; pH 7.4). The skin and muscle layer were sutured separately, and frogs allowed to recover for six months before being reselected for surgery. A total of five recoverable surgeries were allowed on each frog prior to a terminal surgery in which a lethal dose of tricaine (0.5%) was administered. All the procedures involved in the use of Xenopus laevis frogs were approved by the Animal Ethics Committee of the University of Sydney (Reference number: 2013/5915). The lobes of ovaries were then separated evenly and incubated with Collagenase A (Boehringer Manheim, Germany). The released oocytes were first washed with OR2 buffer then stored in frog Ringer buffer (ND96).

A microprocessor-controlled micropipette puller (PUL-100, World Precision Instruments, Inc. FL, USA) was used to make micropipettes then RNA pulled up into the micropipette by a positive-displacement using micro-injector (Nanoliter micro-injector, World Precision Instruments, Inc. FL, USA). Oocytes were sorted and healthy ones were injected with 35–50 ng of ρ cRNA unless otherwise mentioned.

#### Two-electrode voltage-clamp electrophysiology

The injected oocytes were incubated for between two and four days after injection with desired RNA. Two-electrode voltage clamp instrument; Geneclamp 500 amplifier (Axon Instrument, Sydney, NSW, Australia) and Chart version 5.5.6 was used to measure the various activities at the receptors expressed by these oocytes. The recording microelectrodes were prepared by pulling using PUL-100 micropipette puller (World precision Instruments, Inc. FL, USA) and filled with 3 M KCl.

#### Data analysis

Current responses were normalized to the maximum GABA-activated current (or other ligand which is stated as required) and expressed as a percentage, which was fitted by least squares to Hill equation (Eq 1). Dose response curves were generated using GraphPad PRISM 5.02 (GraphPad Software San Diego, CA). The responses of sub-maximal agonists tested at ρ1 GABA WT and mutant receptors were normalized by the EC_max_ concentrations of the highest efficacious ligand in order to calculate concentration response curves of these ligands.

$$I=\frac{I_{max}\;{\left[A\right]}^{nH}}{\left({EC}_{50}^{\;nH}+{\left[A\right]}^{nH}\right)}$$ Equation 1

Where I is the current response to a known concentration of agonist, I_max_ is the maximum current obtained, [A] is the agonist concentration, EC_50_ is the concentration of agonist at which current response is half maximal and nH is the Hill coefficient.

The inhibitory concentration curves were generated using GraphPad PRISM 5.02 and IC_50_ values were calculated using Eq 2.

$$I=\frac{I_{max}\;{\left[A\right]}^{nH}}{\left({IC}_{50}^{\;nH}+{\left[A\right]}^{nH}\right)}$$ Equation 2

I is the peak current at a given concentration of agonist, I_max_ is the maximal current generated by the concentration of agonist, [A] is the concentration of GABA, IC_50_ is the antagonist concentration which inhibits 50% of the maximum GABA response and nH is the Hill coefficient.

### Statistical analysis

All statistical calculations are presented as mean ± standard error of the mean (SEM) or as mean (95% confidence intervals (CI)). Student’s t-test was performed to determine the statistical significance of the change in EC_50_ and IC_50_ at ρ1 GABA_C_ WT and mutant receptors, where 0.05 was treated as the point of significance.

## Results and Discussion

### Homology modeling and GABA docking studies with threonine 244

**Fig 2:**
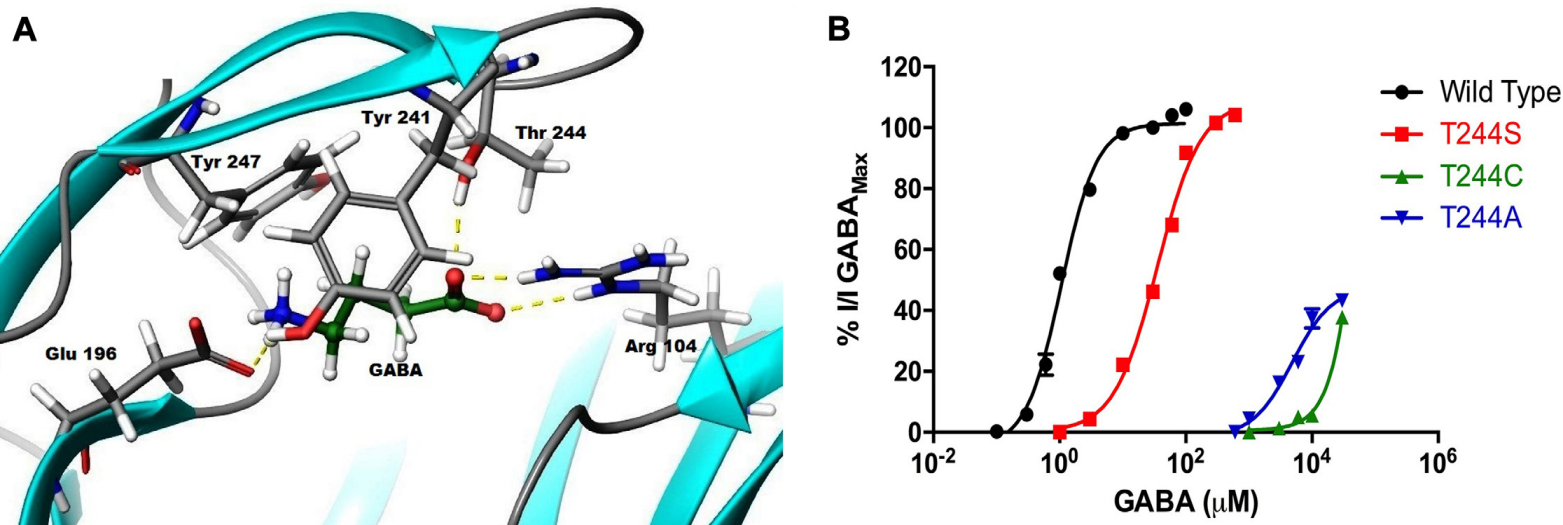
Effect of GABA at mutant receptors. A. GABA and surrounding residues in the orthosteric binding site of the ρ1 GABA_C_ homology model. B. Concentration response curves of GABA at ρ1 GABA_C_ WT, ρ1 T244S, ρ1 T244A and ρ1 T244C receptors, (Data = Mean ± SEM, n = 5). Note: GABA elicited sub-maximal efficacy compared to β-alanine and MTSEA at ρ1 T244A and ρ1 T244C mutant receptors, respectively.

Our generated model predicts a hydrogen bond between the hydroxyl group of the Thr244 in loop C and the carboxylate group of GABA (Fig 2A). This interaction assists in agonist binding and may facilitate gating due to the movement of loop C inward, leading to the open conformation and activating the receptor [27].

### Mutation of threonine 244 into serine, alanine and cysteine

GABA was first tested on all mutant receptors in order to determine the functionality of these receptors. The ρ1 GABA_C_ T244S mutant receptors showed a 35-fold decrease in GABA potency with an EC_50_ of 35 μM as previously reported [21, 22]. ρ1 GABA_C_ T244A and ρ1 GABA_C_ T244C mutant receptors initially appeared to be non-functional or insensitive to GABA at a concentration of 30mM. However, increasing the concentration of mutant RNA injected into the oocytes from 50–100 ng/μl to 300 ng/μl resulted in the expressions of receptors that were weakly responsive to GABA at high concentrations (Fig 2B).

### ρ1 GABA_C_ T244S mutant receptors

The moderate decrease in GABA potency that occurs at ρ1 T244S mutant receptors suggests the importance of the threonine methyl group in restricting the position of the hydroxyl group which is predicted by modeling to form a hydrogen bond with the carboxylate group of GABA [27]. When the partial agonists imidazole-4-acetic acid and muscimol were tested at ρ1 T244S mutant receptors, their agonist effects were eliminated by the mutation and they acted only as competitive antagonists. Although the actions of agonists and partial agonists were affected by the T244S mutation, the activity of the competitive antagonists in this study remained unchanged at these mutant receptors compared to ρ1 WT receptors [22].

#### Agonist effects of TACA and CACA at ρ1 T244S receptors

**Fig 3:**
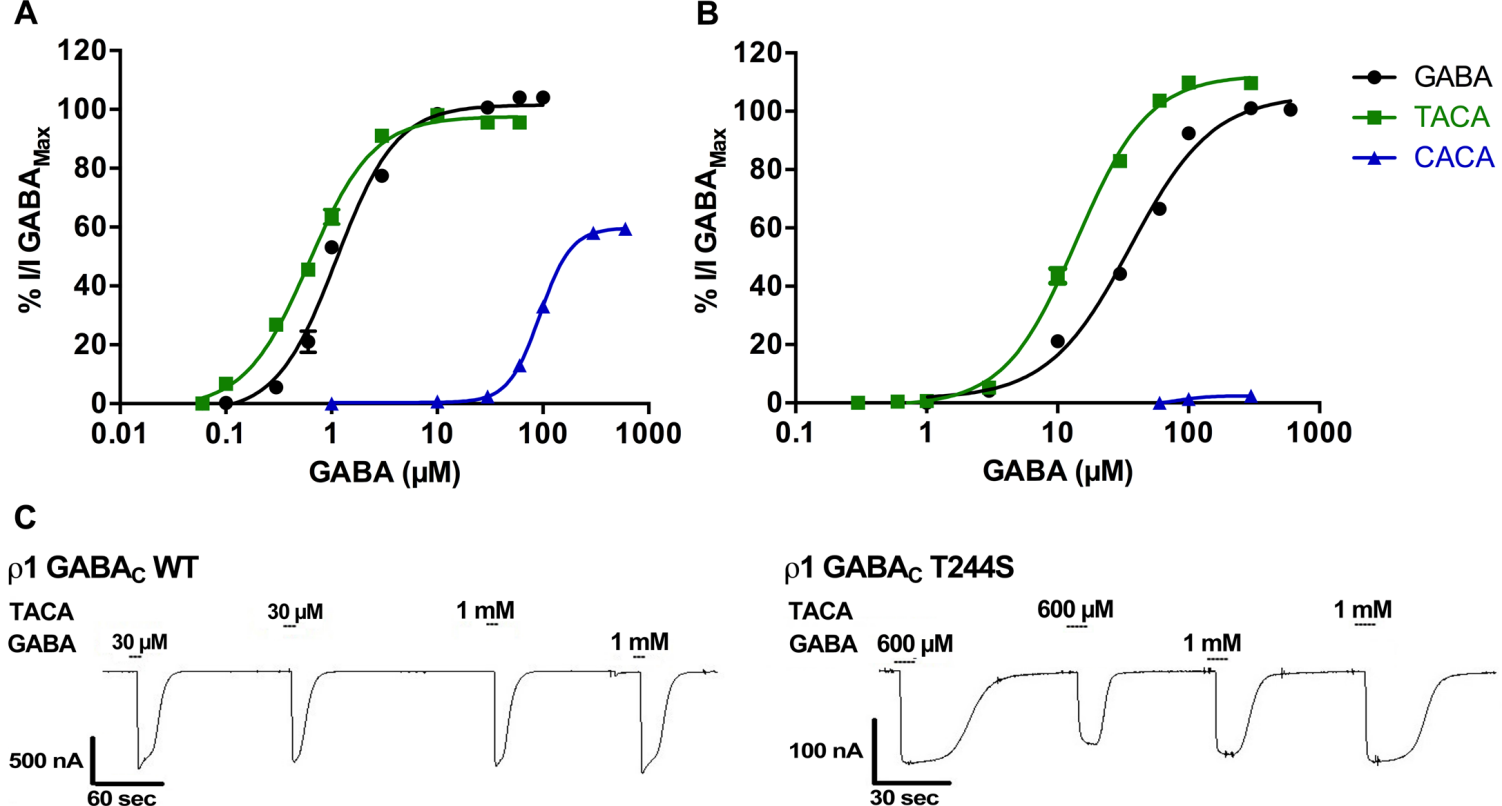
Effect of conformational restriction on the efficacy of ligands at GABA receptors. Concentration response curves of GABA and the unsaturated analogues, TACA and CACA at (A) ρ1 WT and (B) ρ1 T244S receptors, (Data = Mean ± SEM, n = 5). (C) Sample traces of the maximal responses of GABA and TACA at ρ1 WT and ρ1 T244S receptors.

The *trans* and *cis* isomers of crotonic acid, TACA and CACA respectively (Fig 1), are conformationally restricted unsaturated GABA analogues. TACA a potent agonist (EC_50_ = 0.6 μM) at ρ1 WT receptors (Fig 3A), showed a 25-fold decrease in activity at ρ1 T244S mutant receptors (EC_50_ = 14 μM, Fig 3B). However, the partial agonist CACA (EC_50_ = 95 μM) at ρ1 WT receptors (Fig 3A) became almost inactive at ρ1 T244S mutant receptors, resulting in only a minimal response at 300 μM (Fig 3B).

**Fig 4:**
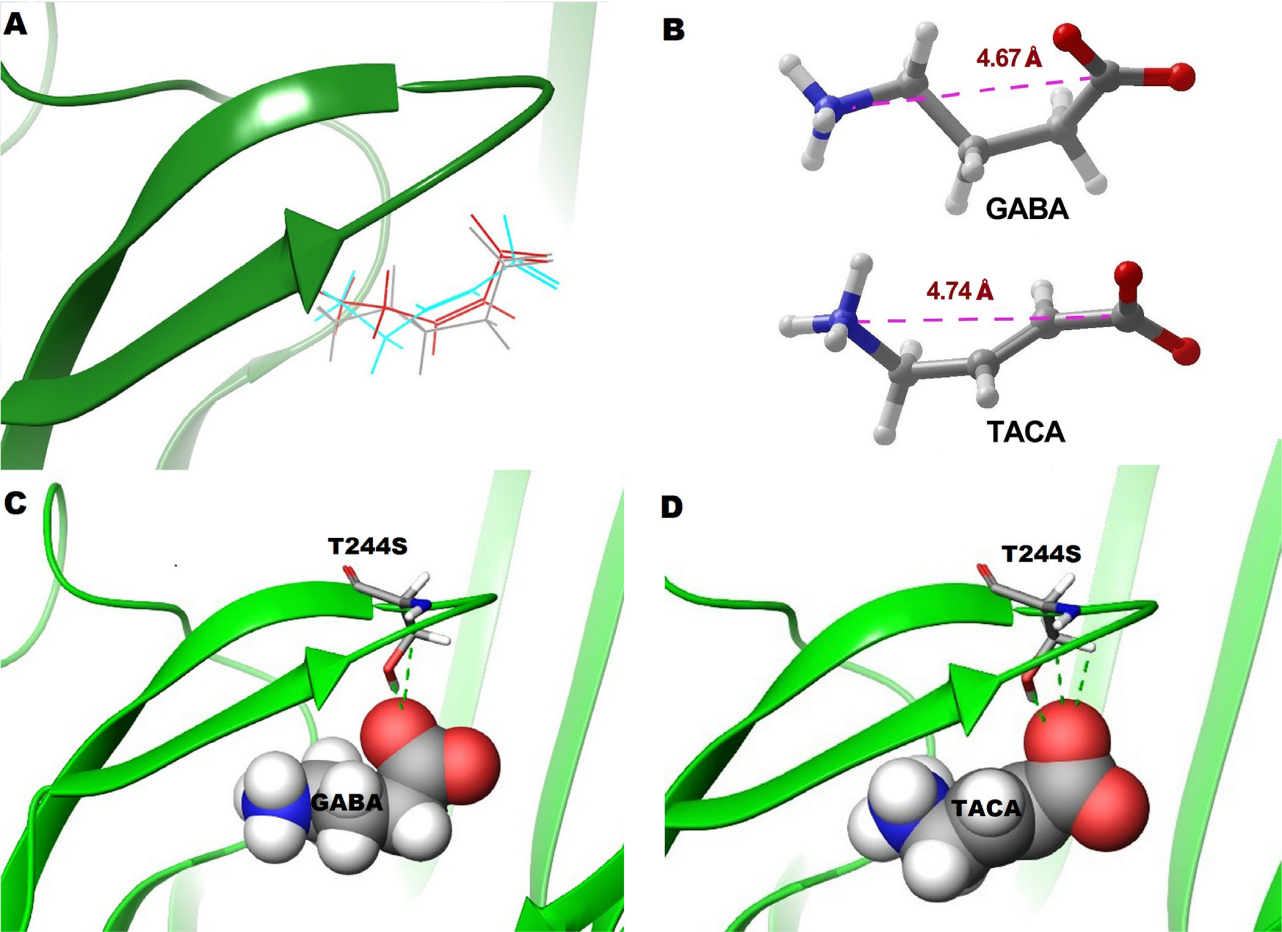
Characterisation of response and binding of GABA and TACA at ρ1 WT and ρ1 T244S receptors. (A) GABA, TACA and CACA docked in the orthosteric binding site of ρ1 GABA_C_ homology model based on GluCl in open conformation. (B) Poses of GABA and TACA in their binding conformations showing distances between the two poles. (C) Various contacts of the side chain of GABA with the side chain of serine at Thr244 site. (D) Various contacts of the side chain of TACA with the side chain of serine at Thr244 site. The rotamer of serine shown in D and E has similar Chi1 and Chi2 (i.e. first and second dihedral angles of the side chain) values to the predicted conformation of Thr244 at this site (*i*.*e*. Chi = 91 and Chi2 = –179).

The efficacy of the unsaturated ligands was also altered as a result of the ρ1 T244S mutation. Interestingly TACA, which shows 95% efficacy relative to the GABA maximal response at ρ1 WT receptors is slightly more efficacious (110%) than GABA at ρ1 T244S receptors (P ˂ 0.05) (Fig 3C). CACA which shows only 60% efficacy relative to GABA maximal response at ρ1 wild type receptors has only 2% efficacy at ρ1 T244S receptors (Fig 4A).

TACA has a slightly longer distance between the carboxylate and ammonium poles than GABA (Fig 4B), and our model predicts the bound orientation of the carboxylate adopting a slightly different orientation to that of GABA and CACA (Fig 4A and 4B) and is predicted to be more able to form contacts with the side chain of the serine residue at position 244 (Fig 4C and 4D). This interaction may further stabilize the open conformation and be responsible for the higher efficacy of TACA at these mutant receptors.

#### Effects of CACA co-applied with GABA EC_50_ at ρ1 T244S receptors

**Fig 5:**
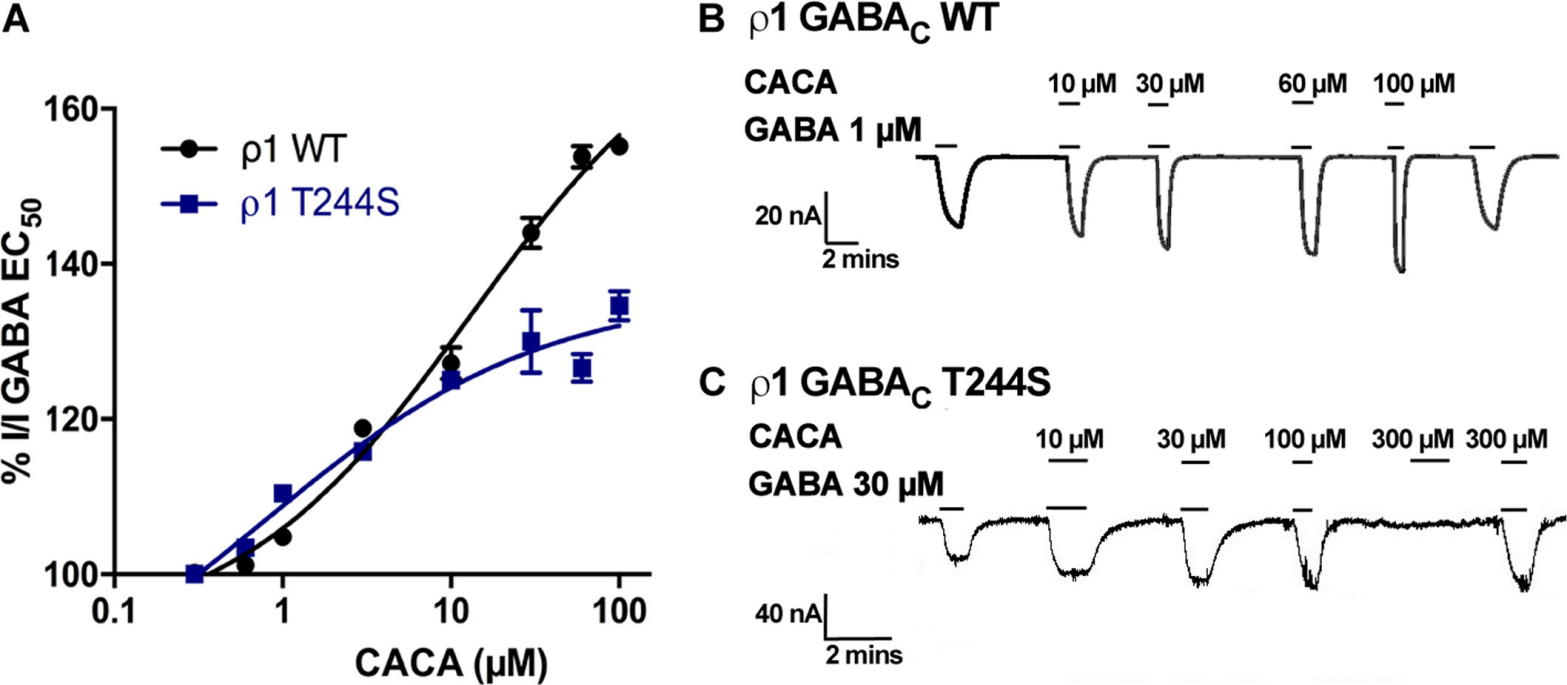
Enhancement of the GABA EC_50_ response by CACA at ρ1 WT and ρ1 T244S receptors. (A) Concentration response curve of the co-application of increasing concentrations of CACA in the presence of GABA EC_50_ at ρ1 WT and ρ1 T244S receptors, (Data = Mean ± SEM, n = 5). Sample traces of CACA co-applied with GABA EC_50_ at ρ1GABA_C_ (B) WT and (C) ρ1 T244S receptors.

The co-application of CACA has an additive effect on GABA EC_50_ responses at ρ1 WT receptors, reaching up to 150% of GABA EC_50_ efficacy at 100 μM CACA (Fig 5A and 5B). The additional effect of CACA is similar in magnitude to the effect of CACA applied alone at ρ1 WT receptors. There are five equivalent binding sites and it has been predicted that at least three molecules of GABA are required to bind at each pentameric GABA_C_ receptor in order to elicit a response. These results suggest that CACA is binding at vacant sites on the receptors activating the receptor so that more individual receptors are open, leading to a maximal response.

Interestingly, despite the fact that CACA applied alone is almost inactive at ρ1 T244S receptors (Fig 5B), CACA applied in the presence of GABA EC_50_ shows an additive effect on GABA EC_50_ responses at these mutant receptors, The magnitude of 300 μM CACA applied alone is less than 2% of GABA I_MAX_, however the GABA EC_50_ effect is enhanced by approximate 20% at 100 μM CACA (Fig 5). This suggests that binding and/or activation by GABA changes the conformation of the vacant binding sites allowing CACA activate the receptor more effectively that when CACA is applied alone, leading to an additive effect.

#### Agonist effects of glycine, β-alanine and 5-aminovaleric acid

**Fig 6:**
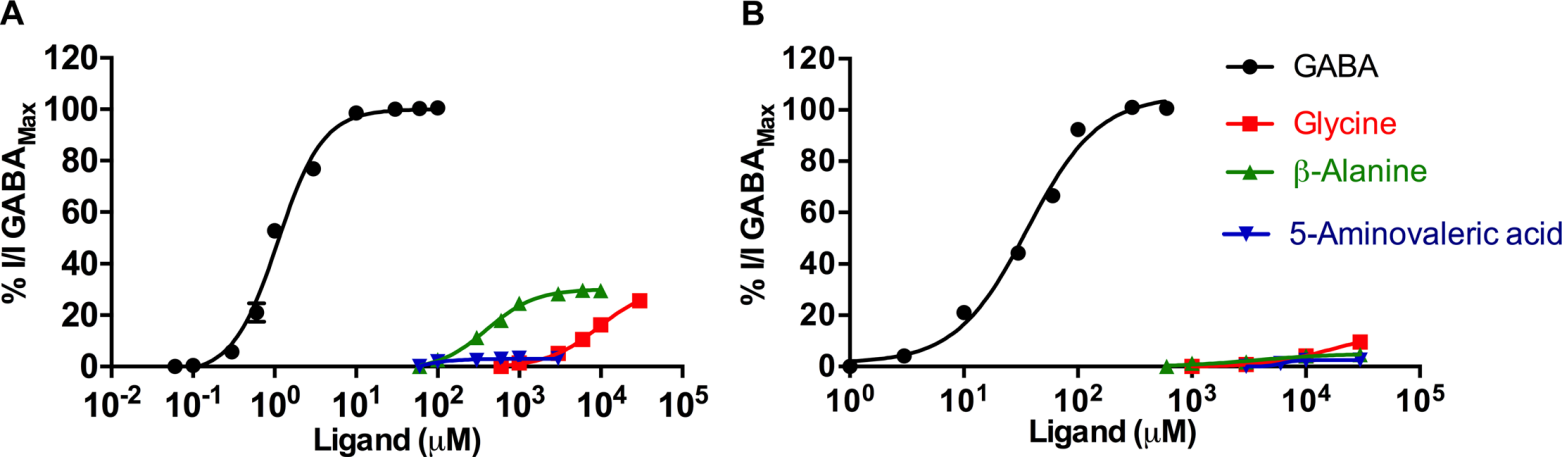
Activity of GABA, glycine, β-alanine and 5-aminovaleric acid at ρ1 WT and ρ1 T244S receptors. Concentration response curves of GABA, glycine, β-alanine and 5-aminovaleric acid at ρ1 WT (A) and ρ1 T244S (B) receptors, (Data = Mean ± SEM, n = 5).

At ρ1 WT receptors, the analogues of GABA with varying numbers of carbon atoms in the backbone, glycine, β-alanine and 5-aminovaleric acid, respectively (Fig 1) are weak partial agonists (Fig 6A) [37, 38]. Compared to their effects on WT receptors, the potencies and efficacies of these partial agonists were decreased at ρ1 T244S mutant receptors. Both glycine and β-alanine showed significant decreases in efficacy, with a maximum of 8% and 5% relative to the GABA maximal response at 30 mM. The least efficacious compound at ρ1 WT receptors, 5-aminovaleric acid showed a large decrease in potency, shifting the curve to the right, however the ligand was still able to weakly activate the mutant receptors at very high concentrations (6000μM, 3% efficacy relative to the GABA maximal response at 30 mM) (Fig 6B).

#### Effects of glycine, β-alanine and 5-aminovaleric acid co-applied with GABA EC_50_ at ρ1 T244S receptors

**Fig 7:**
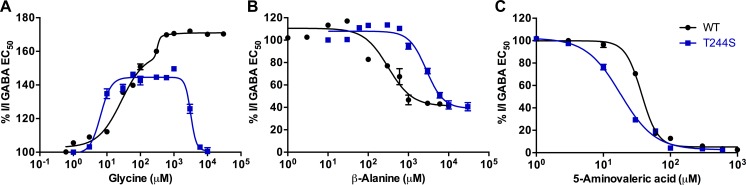
Effect of glycine, β-alanine and 5-aminovaleric acid on the GABA EC_50_ response. Concentration response curves of GABA EC_50_ in the presence of (A) glycine, (B) β-alanine and (C) 5-aminovaleric acid at ρ1 WT and ρ1 T244S receptors, (Data = Mean ± SEM, n = 5).

The partial agonist glycine displays an additive effect on the GABA EC_50_ responses at ρ1 WT receptors, 180% efficacy (relative to the GABA EC_50_ response set to 100%), and has a similar effect at ρ1 T244S mutant receptors but with less magnitude, 145% efficacy. At ρ1 T244S receptors this additive effect is reduced at concentrations higher than 1 mM (Fig 7A). Despite only being able to directly activate these receptors by 25% (ρ1 WT) and 2% (ρ1 T244S) at mM concentrations (Fig 7A), the significant additive effects of glycine at both ρ1 WT and T244S receptors suggests that glycine is able to bind at vacant binding sites and is able to more effectively activate these receptors. Glycine molecules are not able to effectively compete with GABA at sites where GABA is bound at ρ1 WT receptors. This is most likely because GABA has a much higher binding affinity at ρ1 WT receptors. However, at ρ1 T244S mutant receptors the binding affinity of GABA is likely to be reduced and, therefore at very high concentrations of glycine, some competition between the two ligands for the same binding site may occur, but without significant displacement of GABA molecules, resulting in fewer channels being activated and, therefore the additive effect of glycine is diminished at 10mM (Fig 7A).

At concentrations of up to 100 μM β-alanine in the presence of GABA EC_50_ has weak additive effects at ρ1 WT receptors (125%) and ρ1 T244S mutant receptors (115%), respectively. At concentrations of greater than 100 μM β-alanine, acts as an antagonist at both ρ1 WT and ρ1 T244S receptors (β-alanine inhibits 60% of GABA EC_50_ at both receptors). However the IC_50_ of β-alanine is increased 8-fold at ρ1 T244S receptors compared to ρ1 WT (120μM and 1 mM of β-alanine antagonize GABA EC_50_ responses at ρ1 WT and ρ1 T244S receptors, respectively) (Fig 7B).

**Fig 8:**
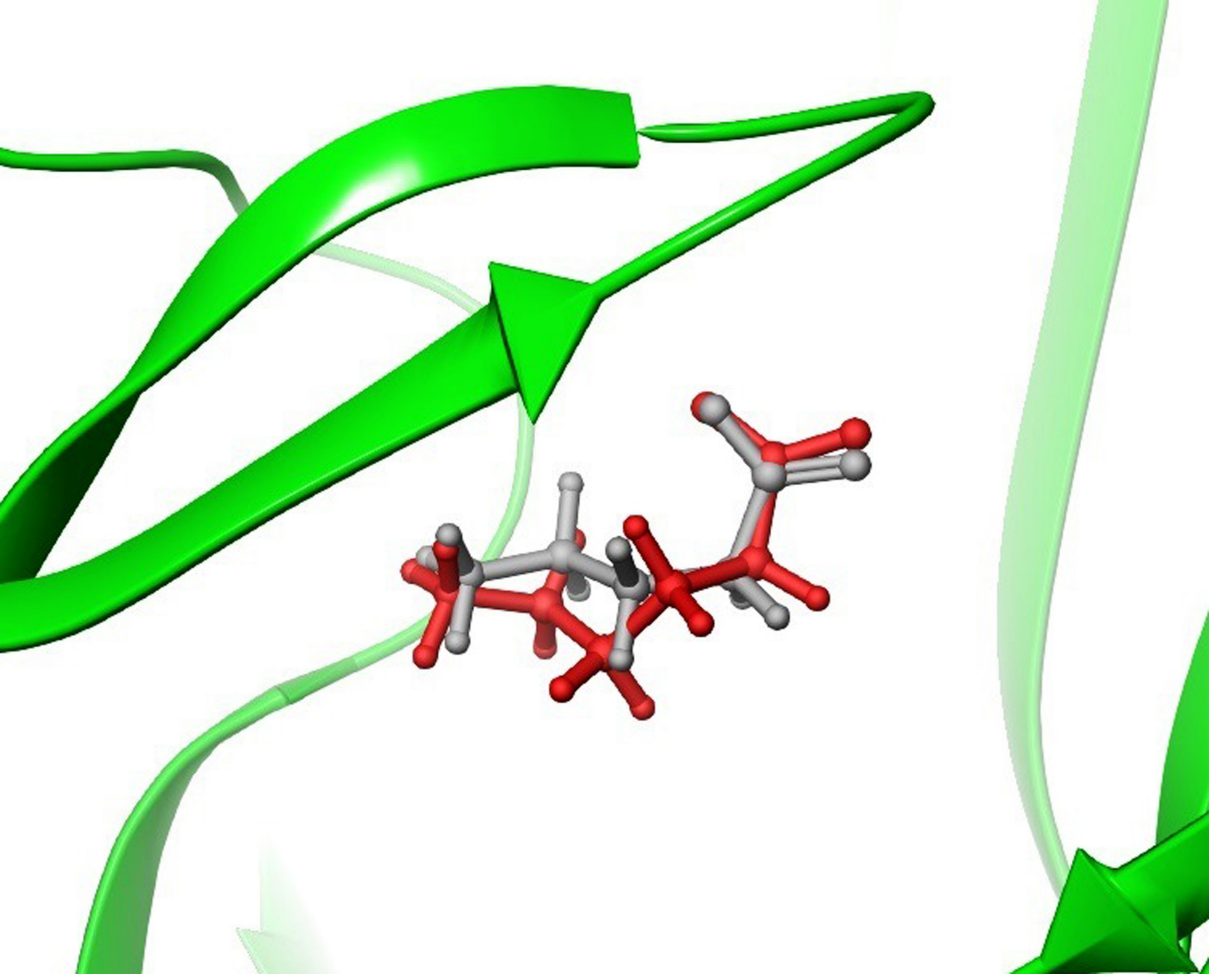
5-Aminovaleric acid fits in the ρ1 GABA_C_ orthosteric binding site in a folded conformation. GABA (white) and 5-aminovaleric acid (red) docked in the orthosteric binding site of ρ1 GABA_C_ homology model based on GluCl in *apo* state.

In the presence of GABA EC_50,_ 5-aminovaleric acid acts as an antagonist at both ρ1 WT and ρ1 T244S receptors at concentrations of greater than 1 μM (Fig 7C)_._ However, the potency of 5-aminovaleric acid is increased two-fold ρ1 T244S receptors with an IC_50_ = 17 μM compared to 37 μM at ρ1 WT receptors (Fig 7C). The extended chain length of 5-aminovaleric acid compared to GABA permits 5-aminovaleric acid to form required interactions with the receptors in the *apo* state and the protein is predominantly not required to undergo a conformational change activating either ρ1 WT nor ρ1 T244S receptors (Fig 8). The additional methylene group of 5-aminovaleric acid may result in further hydrophobic contacts in the binding site that further stabilize the *apo* state over open conformation. The increase in antagonist potency of 5-aminovaleric acid at ρ1 T244S receptors is possibly due to the extended chain length resulting in increased flexibility allowing the carboxylate group to form interactions with the side chain of serine at position 244 more effectively than GABA.

### Isoguvacine and ρ1 T244S mutant receptors

#### Agonist effect of isoguvacine

**Fig 9:**
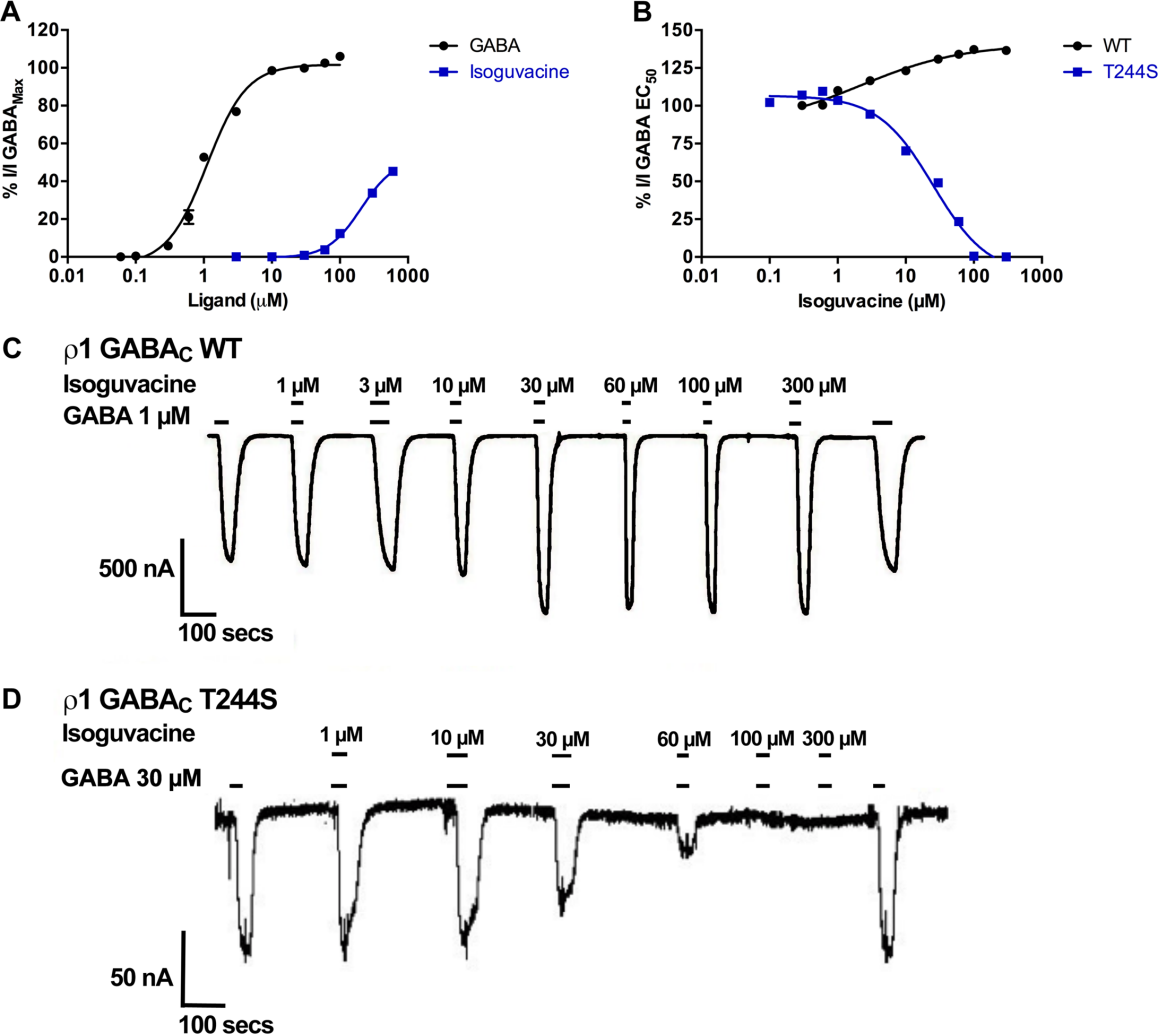
Characterisation of the effect of isoguvacine at ρ1 T244S mutant receptors. (A) Concentration response curves of GABA and isoguvacine at ρ1 WT receptors, (Data = Mean ± SEM, n = 5). (B) Concentration response curves of the co-application of isoguvacine with GABA EC_50_ at ρ1 GABA_C_ WT and ρ1 T244S receptors, (Data = Mean ± SEM, n = 5). Sample response traces of isoguvacine when co-applied with GABA EC50 at (C) ρ1 GABAC WT receptors and (D) at ρ1 T244S receptors.

The heterocyclic GABA analogue; isoguvacine (Fig 1) was tested at ρ1 WT and ρ1 T244S receptors. This ligand is weak partial agonist, activating WT receptors with an EC_50_ = 200 μM [39] and 45% efficacy relative to the GABA maximal response. However, at ρ1 T244S mutant receptors the potency and efficacy of isoguvacine are significantly reduced with only a 30% response elicited at a concentration of 600 μM (Fig 9A).

**Fig 10:**
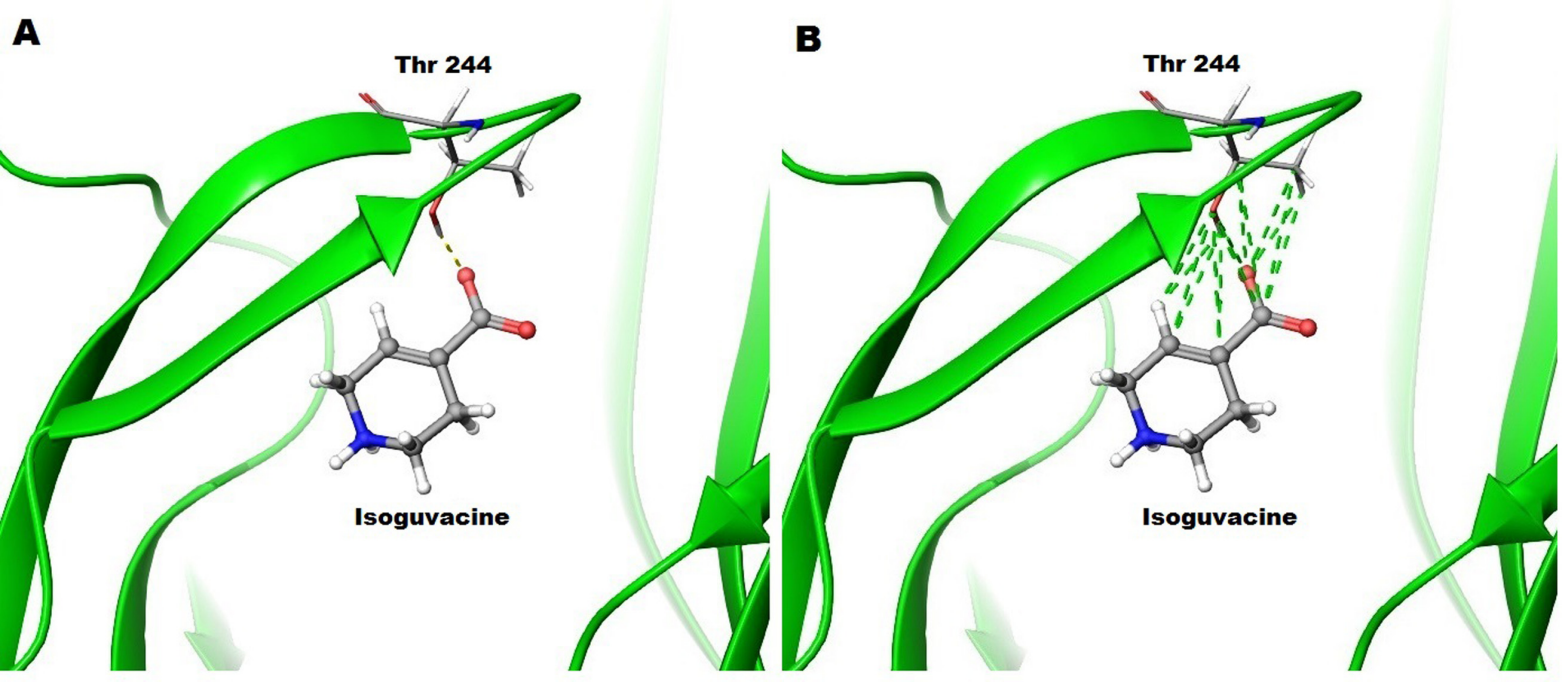
Docking studies of isoguvacine in the orthosteric binding site of ρ1 GABA_C_ homology model. (A) H-bonds and (B) hydrophobic interactions predicted to be formed by isoguvacine and the ρ1 GABA_C_ receptor.

Docking studies of this ligand in the ρ1 GABA_C_ homology model predict H-bonding of the hydroxyl group of Thr244 with the carboxylate group of isoguvacine (Fig 10A). There are also hydrophobic interactions between the side chains of Thr244 and isoguvacine (Fig 10B). These interactions may possibly further stabilize the receptor in open conformation. The significant reduction in potency and efficacy of isoguvacine when serine is introduced at the Thr244 site suggests the importance of the H-bond and other interactions between side chain of isoguvacine and Thr244 in stabilizing the receptor in open conformation.

#### Antagonist effects of isoguvacine

At ρ1 WT receptors isoguvacine has a moderate additive effect on the GABA EC_50_ response of up to 135% (Fig 9B and 9C). However, at ρ1 T244S mutant receptors isoguvacine acts as a full antagonist (IC_50_ = 25.3 μM) against GABA EC_50_ (Fig 9B and 9D).

These results suggest that isoguvacine alone binds to ρ1 T244S mutant receptors but does not stabilize the open conformation. In the presence of GABA EC_50_ the effect of isoguvacine is changed from a additive effect at ρ1 WT receptors to antagonist ρ1 T244S mutant receptors. Removal of the methyl group from the side chain allows serine to adopt an orientation that permits the formation of an H-bond with isoguvacine that does not require the movement of loop C in the open conformation.

Studying the activity of structurally different partial agonists at ρ1 GABA_C_ T244S mutant receptors indicates that the overall effect of the mutation is determined by the structure of the ligand. The efficacy and potency of the aliphatic partial agonists (glycine, β-alanine and 5-aminovaleric acid) decreased significantly however they were still able to weakly activate ρ1 T244S receptors at very high concentrations. However, the heterocyclic partial agonist, isoguvacine, which lacks conformational flexibility, was converted to full antagonist.

### Effect of the T244S mutation on the activity of the antagonists TPMPA and THIP

Compared to the effect of the GABA_C_ antagonists, TPMPA and THIP (Fig 1) on WT receptors, the T244S mutation has no effect on the potencies of these ligands [40, 41]. Modeling and docking studies with the GABA_C_ model based on GluCl in the *apo* state predicts that loop C is sterically prevented from moving inward and forming a shield above the ligand as an essential step in the open conformation. The bulkier structure of the antagonists may inhibit the movement of loop C closer to the ligand preventing the necessary initial step for channel activation [27].

### ρ1 GABA_C_ T244A mutant receptors

**Fig 11:**
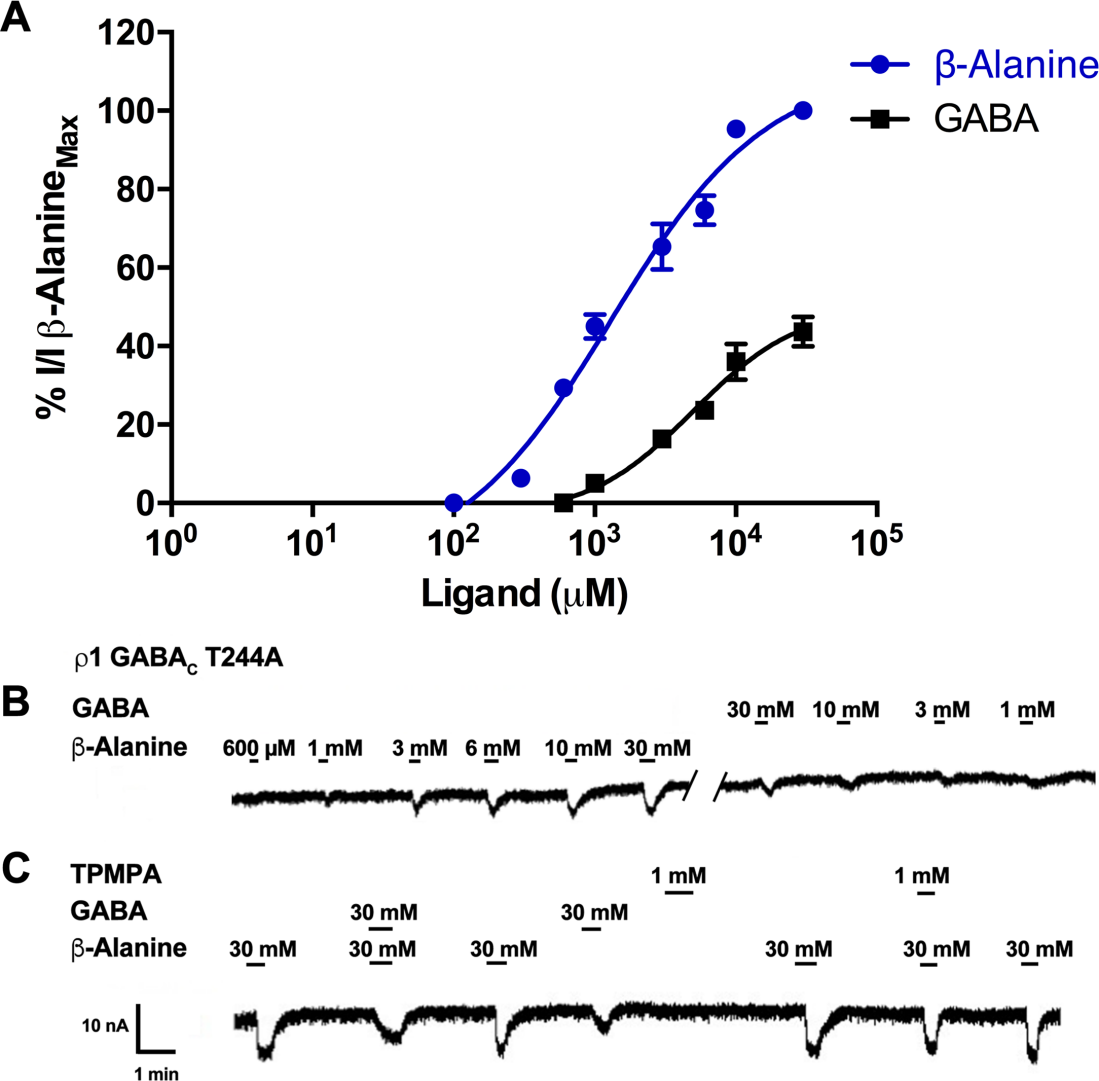
β-alanine is more potent than GABA at ρ1 T244A receptors. Concentration response curves of β-alanine and GABA at ρ1 T244A mutant receptors, (Data = Mean ± SEM, n = 15). (B) Sample response traces of β-alanine and GABA at ρ1 T244A mutant receptors. (C) Sample response traces of β-alanine, GABA and TPMPA at ρ1 T244A receptors.

Substitution of alanine for threonine at position 244 removes the hydroxyl group. At ρ1 T244A receptors, both the potency and efficacy of GABA (Fig 11A and 11B) was significantly reduced (ρ1 WT = 1 μM, ρ1 T244A = 4960 μM). The ρ1 WT agonists, TACA and muscimol and partial agonists, glycine and 5-aminovaleric acid were found to be less efficacious than GABA. However, β-alanine with one carbon less than GABA, was found to be more potent (EC_50_ = 1496 μM, Fig 11 A) and approximately two times more efficacious than GABA at a 30 mM concentration on ρ1 T244A mutant receptors (Fig 11B). Compared to its effects on ρ1 WT receptors (Fig 6), the potency of β-alanine was reduced only two-fold at T244A receptors.

**Fig 12:**
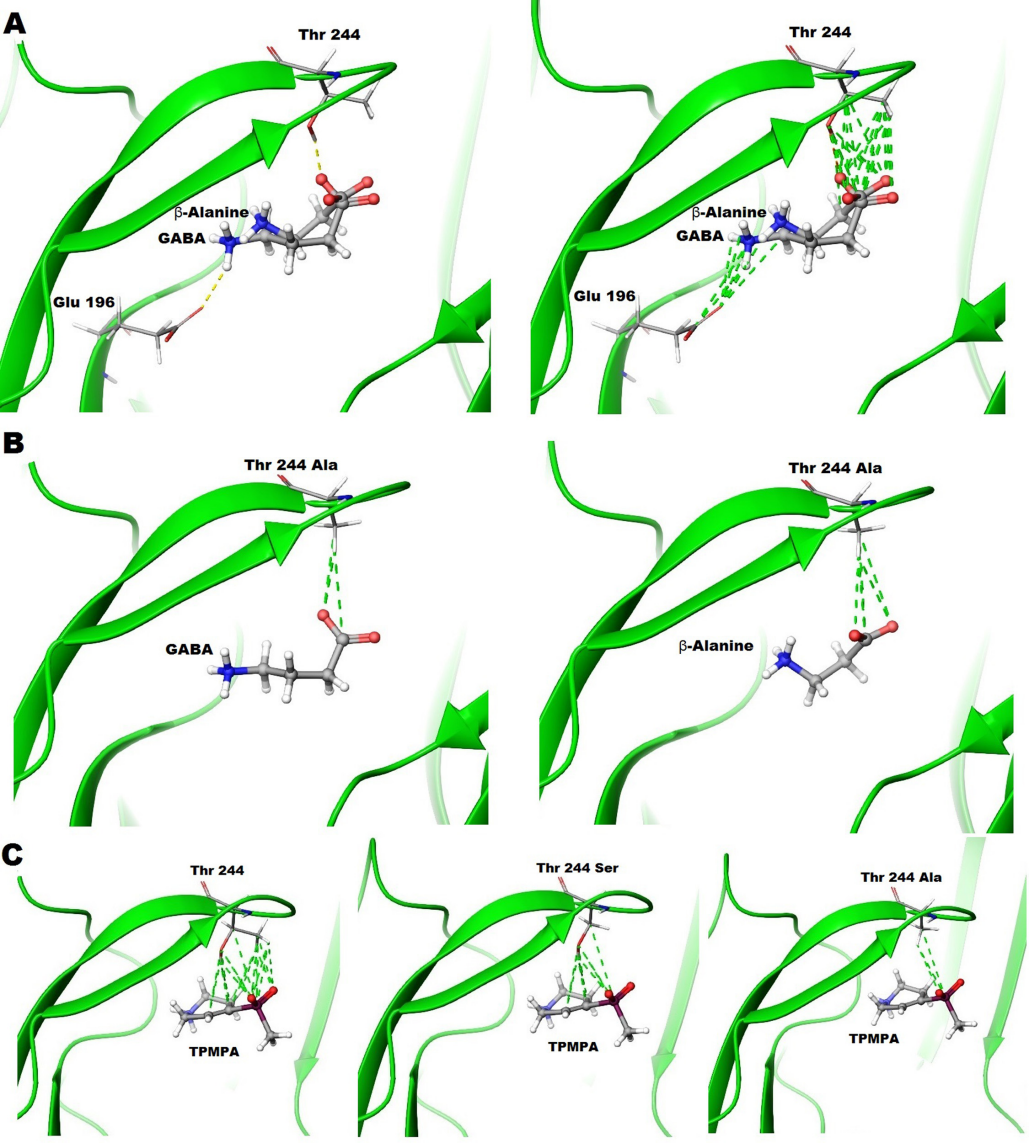
Docking studies showing interactions between ligands and ρ1 receptors. (A) GABA and β-alanine docking studies in the orthosteric binding site of GABA_C_ homology model based on GluCl in open conformation. (Left) hydrogen bonds between the side chains of GABA and β-alanine with side chains of Thr244 and Glu196 residues. (Right) various hydrophobic interactions between the side chains of GABA and β-alanine with side chains of Thr244 and Glu196. (B) GABA and β-alanine docking studies in the orthosteric binding site of GABA_C_ homology model based on GluCl in open conformation. (Left) hydrophobic interactions between GABA side chain and alanine residues at the site of Thr244. (Right) hydrophobic interactions between β-alanine side chain and alanine residue at the site of Thr244. (C) TPMPA docking studies in the orthosteric binding site of GABA_C_ homology model based on GluCl in *apo* conformation. (Left) hydrophobic interactions between the side chain of TPMPA and the side chain of Thr244. (Middle) hydrophobic interactions between the side chain of TPMPA and the side chain of serine residue at the site of Thr244. (Right) hydrophobic interactions between the side chain of TPMPA and the side chain of alanine residue at the site of Thr244.

Our model indicates that β-alanine does not form a salt bridge between its ammonium terminal and Glu196 in same manner as GABA. The ammonium group of β-alanine may be stabilized by the aromatic box in the binding site (Fig 12A). This may also explain the mobility of β-alanine in the binding site compared to GABA, allowing it to move slightly closer to and form more hydrophobic contacts between the alanine of T244A receptors, stabilizing the open conformation (Fig 12B).

Threonine 244 was also mutated to alanine *in silico* in the ρ1 GABA_C_ homology model that was used for docking studies with GABA and β-alanine. The docking studies predict more hydrophobic interactions of the carboxylate group of β-alanine shows with the alanine residue at position 244, than the carboxylate group of GABA. These hydrophobic interactions may provide additional stabilization to β-alanine in the binding site during gating, which may also enhance efficacy and potency of the ligand (Fig 12B). The greater number of interactions of β-alanine with the alanine residue at position 244 may stabilize loop C closer to ligand, which is an essential for channel activation.

The antagonist effects of GABA and TPMPA against the β-alanine response (30 mM) at ρ1 T244A receptors were also investigated. When 30 mM of GABA was co-applied with 30 mM of β-alanine, GABA moderately inhibits the responses elicited by β-alanine (Fig 11C). These results suggest that GABA is acting as partial agonist at ρ1 T244A mutant receptors. On the other hand, the antagonist TPMPA has no effect when applied alone, but weakly antagonizes β-alanine responses at 1 mM (Fig 11C). However, the potency of TPMPA was significantly decreased at the mutant receptors in comparison with ρ1 WT receptors where it has an IC_50_ = 2.3 μM against GABA, indicating that the free hydroxyl of either a serine or threonine at the 244 position is important for the binding and thus antagonist potency of TPMPA. Our modeling studies predict that TPMPA forms the greatest number of hydrophobic contacts with Thr244, while it forms a lower number of hydrophobic contacts with a serine at position 244, and the least number of hydrophobic contacts between TPMPA and the alanine at T244A receptors (Fig 12C).

### ρ1 GABA_C_ T244C mutant receptors

**Fig 13:**
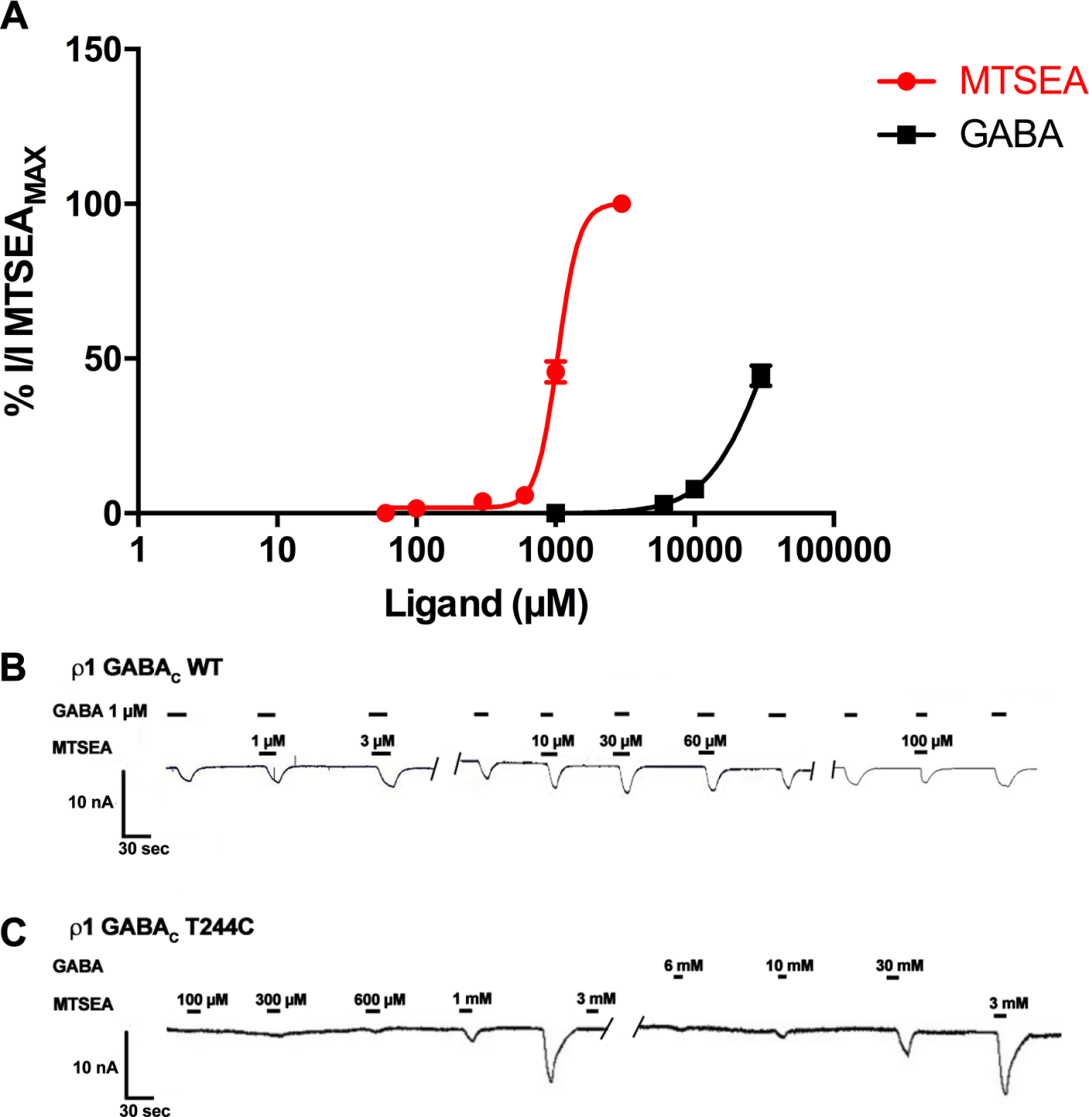
MTSEA produces a reversible response at ρ1 T244C receptors. (A) Concentration response curves of MTSEA and GABA at ρ1 T244C mutant receptors, (Data = Mean ± SEM, n = 15). (B) Sample response traces of MTSEA and GABA at ρ1 WT receptors. (C) Sample response traces of MTSEA at ρ1 T244C receptors.

Initial experiments suggested that T244C mutant receptors did not express, however injection of five times the concentration of RNA normally injected resulted in very weak response to GABA (30 mM). Interestingly, the thio-reactive GABA analogue; 2-aminoethylmethane thiosulfonate (MTSEA) was found to elicit reversible responses at ρ1 T244C mutant receptors with greater potency and efficacy than GABA (Fig 13A and 13B).

MTSEA activates ρ1 WT receptors with very weak efficacy and potency (Fig 13C). When this ligand is co-applied with GABA EC_50_, at concentrations below 100 μM it has weak additive effects to the GABA EC_50_ response, but at concentrations of 100 μM and above it weakly antagonizes the GABA EC_50_ responses (Fig 13B).

**Fig 14:**
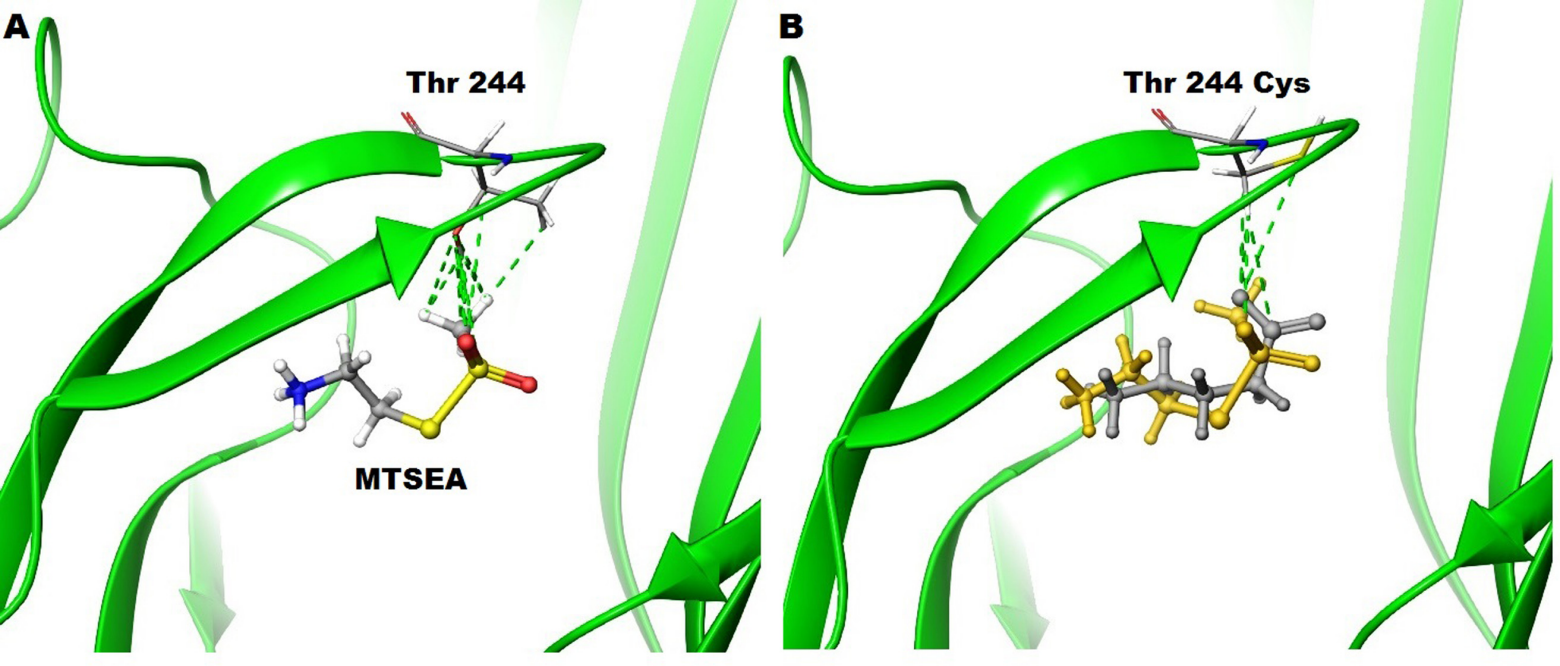
**Docking studies of MSTEA and GABA** (A) in the WT ρ1 GABA_C_ homology model based on GluCl in open conformation. (B) Docking studies of GABA (white) and MTSEA (yellow) with ρ1 T244C homology model based on GluCl where thiol group of Cys244 is oriented away from the binding site (Chi1 = -64°).

Docking studies of MTSEA in the orthosteric binding site of ρ1 homology model predicts that a H-bond does not form between the sulfonyl oxygen of MTSEA and hydroxyl group of Thr244, Although a number of interactions occur between the side chains of MTSEA and Thr244 (Fig 14A). Moreover, replacing threonine with cysteine at position 244 *in silico* predicted that the introduced cysteine exists predominantly as a rotamer in which the thiol is pointed away from the binding site (Chi1 = -64°) which may explain why an irreversible S-S bond does not form between MTSEA and cysteine (Fig 14B). However, MTSEA is still able to activate ρ1 T244C receptors with a similar magnitude to ρ1 WT receptors, although GABA sensitivity at the ρ1 T244C mutant receptors is significantly decreased.

Additionally, due to the methyl group of the methane thiosulfonate, MTSEA is predicted by docking studies to form slightly more interactions with the cysteine residue at the 244 position than the carboxylate group of GABA. Therefore, once the hydroxyl of threonine has been removed, MTSEA may confer better stability of the open channel conformation through hydrophobic interactions at the ρ1 T244C mutant receptors compared to GABA (Fig 14B).

### Threonine 244 and channel activation

GABA in the orthosteric binding site of ρ1 GABA_C_ forms a H-bond with the hydroxyl group of Thr244, and this interaction requires Loop C to move towards GABA. A competitive antagonist essentially stabilizes the closed conformation holding the receptor in the closed conformation and preventing the priming necessary for the channel opening [42], therefore preventing GABA from binding. Those ligands which inhibit agonist responses bind tightly in the site and stabilize the receptor in the inactive state. This is can be achieved in the orthosteric binding site of ρ1 GABA_C_ receptors through antagonists forming additional hydrophobic contacts with aromatic residues that surround the binding site. In addition to blocking the site preventing GABA or other agonists from binding and activating the channel, these interactions stabilize the closed conformation by inhibiting the movement of loop C.

**Fig 15:**
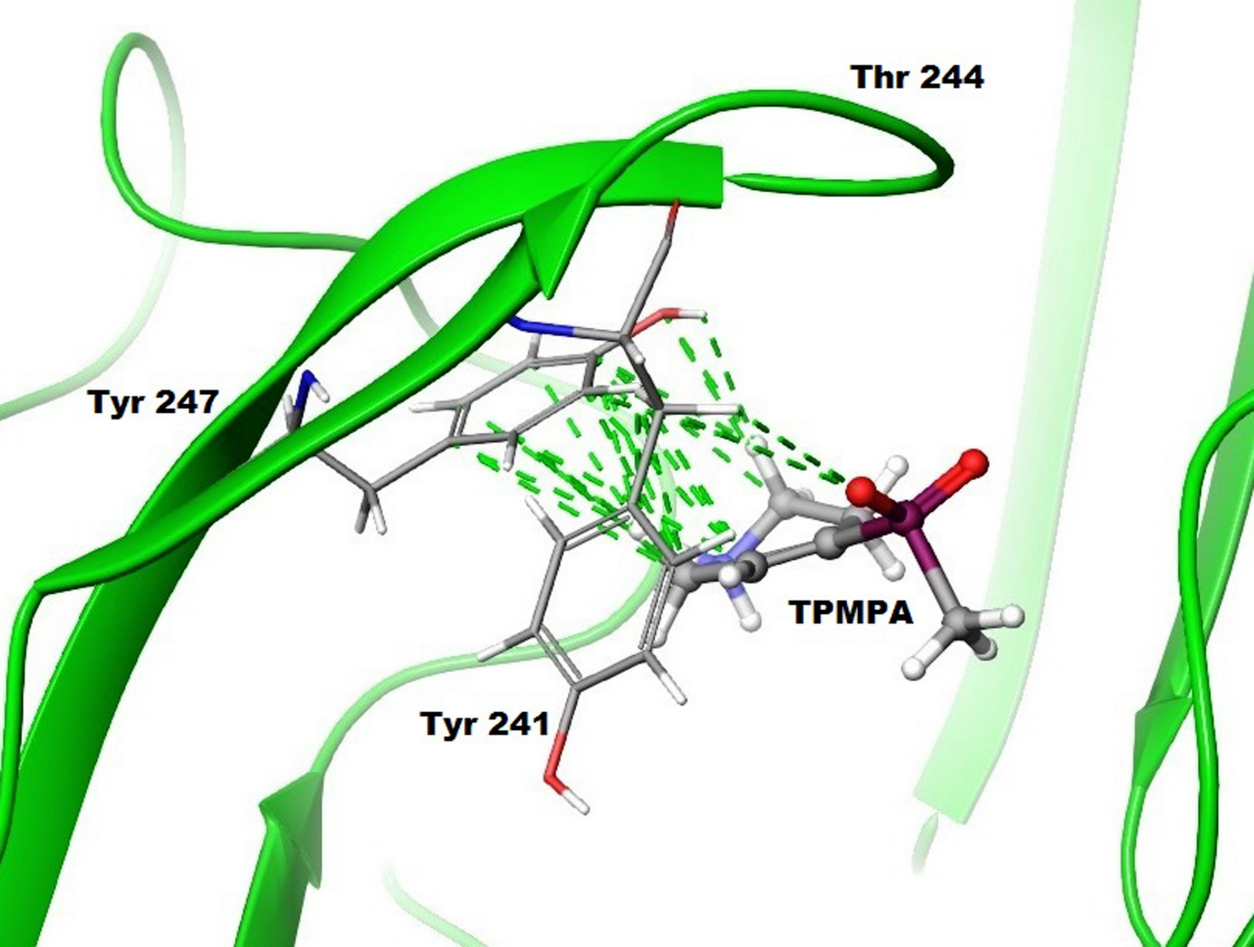
Docking of the antagonist in the ρ1 GABA_C_ homology model based on GluCl in *apo* state. Multiple hydrophobic interactions are formed between TPMPA and the loop C residues Tyr241 and Tyr247.

In the case of ρ1 GABA_C_ ion channels, the bulkier and conformationally restricted heterocyclic ligand, TPMPA binds in the site and forms hydrophobic contacts with Tyr241 and Tyr247 located in the loop C which prevent the loop from moving forward and thus leads to the channel stabilized in close conformation (Fig 15).

## Conclusion

Threonine 244 is a hydrophilic residue with the side chain functional group oriented toward the binding side. GABA docking studies predicted that the hydroxyl group of Thr244 forms an H-bond with carboxylate group of GABA. This residue is also predicted to have various contacts with GABA that may contribute to stability during the gating process. The study of this hydrophilic residue by rational point mutations and testing against GABA, some representative ligands was able to identify the essential role of Thr244 in the closed/open conformations and during channel gating.

The predicted H-bond between the hydroxyl group of Thr244 and the side chain of GABA during the gating process is postulated to require the movement of loop C forward closer to the agonist in the priming step that initiates activation. The efficacy of GABA moderately decreases when serine is introduced at the site. Although GABA is able to activate ρ1 T244A and ρ1 T244C receptors, a greatly increased concentration of RNA was required for expression of these mutant receptors. This suggests that the expression level may have significantly decreased with these mutant receptors. The partial agonists studied demonstrated significant decreases in their potencies and efficacies at ρ1 GABA_C_ T244S mutant receptors. The additive/inhibition effects of these partial agonists were also shown to moderately decrease at the same mutant receptors. However the tested aliphatic partial agonists retained their agonist/antagonist effects while the tested heterocyclic partial agonists became inactive at these mutant receptors, fully antagonizing GABA EC_50_ responses. These results suggest that structural differences of partial agonists may result in the formation of different interactions leading to the different efficacies despite similar structural features. The studied competitive antagonists did not show changes in the potency at T244S mutant receptors in comparison to wild type receptors. The bulkier structure of these antagonists may prevent the movement of loop C as the two aromatic residues (Tyr241 and Tyr247) in loop C could stabilize the ligand in the binding site without moving the loop forward which may confer what model predicted that antagonists aren’t forming the H-bonding with Thr244 (Fig 15). Suggesting both the priming and flipping steps in the early stages of receptor activation appear to be key determinants in the efficacy of an agonist.

In the absence of the hydroxyl side chain at the Thr244 position through the T244A mutation, β-alanine demonstrated the highest efficacy of all agonists and partial agonists tested at ρ1 GABA_C_ T244A mutant receptors, including GABA. Homology model studies predicted that β-alanine forms more contacts with the alanine residue at position 244 than GABA. At ρ1 GABA_C_ T244A receptors, β-alanine exhibited decreased responses when GABA or TPMPA was co-applied with it. These results suggest that TPMPA is binding at the orthosteric binding site of these mutant receptors however a significant decrease in its potency was noticed.

GABA elicited only very small responses while MTSEA with a thiosulfonate side chain demonstrated greater efficacy than GABA at ρ1 GABA_C_ T244C mutant receptors. Docking studies predict that MTSEA is not forming S-S interactions with either cysteine at position 244, or with other residues, which suggests that MTSEA does not form a covalent bond. MTSEA which shows only weak activation and additive/inhibition effects at ρ1 GABA_C_ WT receptors, still has the same magnitude of activation at ρ1 T244C mutant receptors. However the responses to GABA are significantly decreased, and it appears that GABA is unable to stabilize the open conformation as at it does at ρ1 wild type receptors.

In summary, Thr244 is an important residue for channel activation as it forms a H-bond with agonists, initiating the conformational changes required for priming of the receptor and stabilizes the open conformation. The forward movement of loop C to form a shield-like cover over the agonist is the essential initial step for channel gating.
